# Cardiovascular mortality among a cohort of hypertensive and normotensives in Rio de Janeiro - Brazil - 1991–2009

**DOI:** 10.1186/s12889-015-1999-4

**Published:** 2015-07-08

**Authors:** Thiago Luiz Nogueira da Silva, Carlos Henrique Klein, Armando da Rocha Nogueira, Lucia Helena Alvares Salis, Nelson Albuquerque de Souza e Silva, Katia Vergetti Bloch

**Affiliations:** Institute of Studies in Public Health, Federal University of Rio de Janeiro, Rio de Janeiro, Brazil; Department of Epidemiology and Quantitative Methods in Health, Oswaldo Cruz Foundation, National School of Public Health, Rio de Janeiro, Brazil; Clementino Fraga Filho University Hospital, Federal University of Rio de Janeiro, Rio de Janeiro, Brazil; Graduate Program in Medicine - Cardiology, Medical School, Federal University of Rio de Janeiro, Rio de Janeiro, Brazil; Department of Clinical Medicine, Medical School, Federal University of Rio de Janeiro, Rio de Janeiro, Brazil

**Keywords:** Hypertension, Blood pressure, Cardiovascular diseases, Mortality, Survival analysis, Proportional hazards models

## Abstract

**Background:**

Although there is strong evidence of the benefits of antihypertensive treatment, the high prevalence of this important cardiovascular risk factor and its complications, as well as the low control rates of hypertension observed in many studies justify the investigation of these relationships in population studies. The objective was to investigate the ratio of cardiovascular disease mortality between hypertensives (non-treated, controlled and uncontrolled) and non-hypertensives in a cohort of a population sample of adults living in *Ilha do Governador*, Rio de Janeiro state, Brazil, who were classified in a survey conducted in 1991 and 1992 and whose death certificates were sought 19 years later.

**Methods:**

A cohort study was performed on probabilistic linkage between data from an epidemiological study of hypertension performed in *Ilha do Governador*, in Rio de Janeiro, Brazil (1991 to 1992) and data from the Mortality Information System of Rio de Janeiro (1991 to 2009). The survey aimed to estimate the prevalence of hypertension and other cardiovascular risk factors in 1,270 adults aged 20 years or older selected through a probabilistic sampling of households at three economic levels (low, middle and high income). We performed a probabilistic record linkage of these databases and estimated the risk of cardiovascular death using Kaplan-Meier method to plot survival curves and Cox proportional hazards models comparing hypertensive subjects all together, and by hypertension subgroups: untreated, controlled, and uncontrolled hypertensives with non-hypertensive ones.

**Results:**

A total of 170 deaths occurred, of which 31.2 % attributed to cardiovascular causes. The hazard ratio for cardiovascular death was 6.1 times higher (95 % CI 2.7 – 13.7) in uncontrolled hypertensive patients relative to non-hypertensive patients. The hazard ratios for untreated hypertensive and controlled hypertensive patients were 2.7 times (95 % CI 1.1 – 6.3) and 2.1 times (95 % CI 0.38 – 11.5) higher than for normotensive patients, respectively.

**Conclusion:**

The present study demonstrated a higher cardiovascular death risk among hypertensive than among non-hypertensive ones that is not associated uniquely to treatment, because uncontrolled hypertensives demonstrated a greater risk than untreated ones. Although the subgroups of hypertensive individuals were susceptible to changes in their classification over the 19 years of the study, the baseline classification was consistent with a worse prognosis in these individuals.

## Background

Hypertension is a chronic condition with major impacts on cardiovascular morbidity and mortality, particularly in developed and developing countries [1–3].

Due to the high prevalence of hypertension worldwide, this condition is the third leading cause of the global burden of disease, accounting for 64 million disability-adjusted life-years (DALY) lost. It is considered the main risk factor for overall mortality, causing more than seven million deaths worldwide [4].

Several cross-sectional studies conducted in the USA indicated that the prevalence of hypertension increased from 23.9 % in 1988–1994 to 28.5 % in 1999–2000 and, to 29.0 % in the period between 2007 and 2008. Meanwhile, the hypertension control increased from 27.3 % in 1988–1994 to 50.1 % in 2007–2008, mainly among individuals aged > 40 years and Caucasians [5]. In Brazil, the prevalence of self-reported diagnosed hypertension ranges from 15.2 % in Palmas, Tocantins (TO) to 28.7 % in Rio de Janeiro (RJ), according to data from a national telephone survey conducted in 2013 [6].

It is estimated that 40 % of strokes and 25 % of myocardial infarctions could be prevented with proper treatment and blood pressure (BP) control [7]. Hospitalizations related to these conditions and other conditions associated with cardiovascular complications cause a large burden on healthcare services. These cardiovascular-related diseases affect a large part of the population who are not aware of their diagnosis of hypertension and do not properly control their blood pressure levels [8].

Hypertension became a more important risk factor for cardiovascular diseases with the increase in life expectancy. Investigating factors associated with cardiovascular disease mortality contributes to the development of public healthcare policies that aim to improve the quality of life of the population.

Although there is evidence in the literature of the benefits of antihypertensive treatment, the difficulty of controlling blood pressure remains a major public health problem. Hypertensive patients who do not treat hypertension or whose treatment does not control their blood pressure levels have worse cardiovascular risk profile. Studies conducted on samples of the general population allow assessment of the impact of inadequate control of blood pressure levels with effect on cardiovascular mortality rates.

The objective of this study was to analyze the ratio of cardiovascular disease mortality between hypertensive individuals (untreated, controlled, and uncontrolled) and non-hypertensive ones in a cohort of a sample of adults living in *Ilha do Governador*, Rio de Janeiro state, Brazil, who were classified in a survey conducted in 1991 and 1992 and whose death certificates were searched in the mortality information system19 years later.

## Methods

Probabilistic linkage was performed between the databases of an epidemiological survey on hypertension conducted in *Ilha do Governador* in the city of Rio de Janeiro, Brazil, and that of the Mortality Information System (Portuguese acronym: SIM - *Sistema de Informações de Mortalidade*) from 1991 to 2009 in the State of Rio de Janeiro.

### The baseline study on arterial hypertension in Ilha do Governador

The baseline study was a survey conducted in *Ilha do Governador* between July 1991 and May 1992 and aimed to estimate the prevalence of hypertension and other cardiovascular risk factors in adults aged 20 years or older who were selected through a probabilistic sampling of households at three economic levels [9].

### Local and population

The *Ilha do Governador* is a political-administrative region that comprise 15 districts among the 33 regions in which the city of Rio de Janeiro is subdivided.,It is an island located in the *Baía de Guanabara*, with 33.53 km^2^. According to the 1991 census its population accounted for 3.6 % (197,158) of the resident population of the municipality of Rio de Janeiro, including 129,474 adults aged 20 years or older (65.7 %), of which 60,244 (46.5 %) were men and 69,230 (53.5 %) were women. This area of study presents heterogeneous social and economic aspects; a demographic profile very similar to the city of Rio de Janeiro with health facilities of low, medium and high complexity.

### Design sample

The survey was conducted in a three stages cluster sample, from 187 census tracts that formed the *Ilha do Governador*. The sample was stratified according to the average household income of each sector: low-income, middle-income or high income. At the first stage ten census sectors in each of the three socioeconomic stratum (low, middle and high income) were selected by a systematic selection process with probability proportional to the size of each sector. In the second stage 25 households were randomly selected in each of the ten sectors selected at the first stage. In the third stage all subjects aged 20 years or more living in the selected households was selected to compose the final sample [9].

To estimate the sample size it was considered a prevalence 20 % of hypertension, with a sampling error of 2.5 % and a confidence interval of 95 % (bilateral alpha of 2.5 %), considering a reduction of heterogeneity by 1/3 due to clustering effect, resulting in approximately 1,500 individuals.

The same number of households was selected in each stratum, accounting for about 500 individuals in each one (an average of 2.46 adults per household according to census information). Assuming a loss of 10 % of residents in the selected households resulted in a required sample of 250 households in each stratum [9].

The overall effective sample of all three stratum was 674 households (89.9 % of the planned).

### Data collected

Trained interviewers applied an individual questionnaire on demographic information (age, gender, skin color, nationality and marital status); socioeconomic characteristics (education, occupation, labor relations and income); lifestyle (smoking, alcohol consumption, dietary and exercise); previous hypertension diagnosis and antihypertensive treatment and morbidity (history of previous diseases related to the cardiovascular, renal and respiratory systems, use of medications) and reproductive history of women (contraceptive use, pregnancies) [9].

Previously trained and supervised examiners also held measures such as weight and height, radial pulse, arm circumference and blood pressure at home, following a protocol to ensure accuracy and standardization of data collected.

Blood pressure levels were determined with a mercury sphygmomanometer designed to avoid measurement errors. Two blood pressure measures on the same visit were realized in the left arm, with the individual in a sitting position, with an interval of at least 20 min between each one. The second measurement was used to classify the individual as hypertensive or not.

### Mortality Information System

The Health Ministry is responsible for the National Mortality Information System that contains data on mortality throughout the country. It has demographic information such as age, date of birth, gender, skin color, marital status, nationality and place of residence as well as information about death, such as, date and cause of death from the death certificate. Information about the accuracy of coding of death certificates can be found at Jorge *et al.* [10].

### Record linkage

The database linkage was performed using the software Reclink 3 following the steps and parameters recommended by Camargo & Coeli [11]. Reclink 3 is a system for database linkage based on the probabilistic record linkage technique, to match observations between two datasets where no perfect key fields exist. The method consists of standartization of databases and linking using blocking steps comparing the variables “Name/Date of Birth/Sex” to determine a cut-off score [12].

The method had a sensitivity of 85.5 %, specificity of 99.4 %, positive predictive of 98.1 % and negative predictive of 94.9 % using similar databases as in the present study [13].

### Study variables

The cardiovascular causes of death between 1991 and 1995 (obtained from the SIM database) were classified with codes 390 to 459, according to the 9th revision of the International Classification of Diseases (ICD-9), and with codes I00 to I99, according to chapter IX of ICD-10.

The characteristics of the studied population and the proportion of deaths associated with either cardiovascular or non-cardiovascular causes were analyzed. The prevalence of hypertension was estimated and adjusted according to the sampling design with 95 % confidence interval (95 % CI) using the statistical routines for complex samples, Survey (svy), in the program Stata 11.0.

Subjects presenting systolic blood pressure (SBP) lower than 140 mmHg and diastolic blood pressure (DBP) lower than 90 mmHg, without any antihypertensive treatment at the time of the study, were classified as non-hypertensive. Those subjects presenting SBP higher than or equal to 140 mmHg or DBP higher than or equal to 90 mmHg [2, 3] at the second blood pressure measurement as well as those presenting any blood pressure level while on antihypertensive treatment were classified as hypertensive. Treatment was considered as anti-hypertensive drug use.

We classified the hypertensives as:

- Untreated hypertensives: high BP without any treatment at the time of the study.
- Controlled hypertensives: SBP < 140 mmHg and DBP < 90 mmHg who were being treated at the time of the study
- Uncontrolled hypertensives: high BP despite of treatment at the time of the study.

Subjects were considered cigarette smokers or alcohol users when they reported smoking or drinking alcoholic beverages, regardless of frequency. Leisure-time physical activity was classified as present when practiced at least once a week; occasional when practiced less than once per week; or absent when it was never practiced.

For the body mass index (BMI) criteria, subjects were categorized as normal weight with a BMI lower than 25 kg/m^2^ and as overweight with a BMI greater than or equal to 25 kg/m^2^.

Skin color was classified by the interviewer based on the observation of ethnic characteristics.

Income was categorized according to the stratification used in the study design of the Hypertension survey [9]. The subjects’ educational levels were classified into three categories: Illiterate; Intermediate education (primary/secondary education - incomplete or complete primary education, complete secondary education, or incomplete higher education); and Superior education (higher education - completed higher education).

### Analysis

For the survival analysis, the time interval between the date of the subjects’ participation in the survey and the date of cardiovascular death was used. The cumulative probabilities of survival for subjects with hypertension and with cardiovascular disease risk factors were assessed by the Kaplan-Meier method [14] and considered statistically significant when *p* < 0.05 using the log-rank test [15] in the bivariate analysis.

The Cox proportional hazards model [16] and the Survey routine (Stata 11.0) were used for multivariate analysis. The risk factors with *p* < 0.20 obtained in the bivariate analysis and tested in the model as potential confounders. Regardless of statistical significance or impact on the HR (hazard ratio), the variables sex, age and educational level and were kept in the final model because they were associated with both exposure and outcome.

This study was approved by the Research Ethics Committee of the Institute of Studies in Public Health, Federal University of Rio de Janeiro (*Instituto de Estudos em Saúde Coletiva, Universidade Federal do Rio de Janeiro* - IESC-UFRJ) on 15 March 2012 (process number: 6813). The mortality database with identification was released by the health state board of RJ after submission by researchers of a commitment term guaranteeing data security and confidentiality.

## Results

A total of 170 deaths were identified from the probabilistic linkage analysis among the SIM databases from the period between 1993 and 2009 (2,257,984 records) and the *Ilha do Governador* survey database (1,270 records). Because the SIM databases from 1991, 1992 and part of the 1996 were not avaiable, due to technical problems, the death data from these years were obtained from register offices.

The mean age of the sample from *Ilha do Governador* was 43.4 years (SD = 15.5 years). The majority of this sample consisted of women (55.7 %), subjects with white skin (65.3 %) and those who had completed intermediate education (63.3 %). The distribution by sex of the characteristics of the sample of *Ilha do Governador* is shown in Table 1.

**Table 1:** Socioeconomic characteristics, lifestyle and morbidity by sex in the cross-sectional study of *Ilha do Governador*, 1991/92

		Male		Female		Total	
		n	%	N	%	n	%
**Demographic characteristics**	**Age**						
	20 to 39 years	255	45.3	320	45.3	575	45.3
	40 to 59 years	212	37.7	261	36.9	473	37.2
	60 years or older	96	17.0	126	17.8	222	17.5
	Skin color* ^**1**^						
	White	369	66.0	460	65.2	829	65.5
	Black	42	7.5	56	7.9	98	7.8
	Other	148	26.5	190	26.9	338	26.7
**Socioeconomic status**	**Income** ^*2^						
	Low	192	34.1	248	35.1	440	34.7
	Medium	206	36.6	243	34.4	449	35.3
	High	165	29.3	216	30.5	381	30.0
	**Educational level** ^*3^						
	Illiterate	20	3.6	51	7.2	71	5.6
	Intermediate	423	75.1	547	77.5	970	76.4
	Superior	120	21.3	108	15.3	228	18.0
**Lifestyle**	**Cigarette smoking**						
	Never	194	34.5	387	54.7	581	45.8
	Quit	163	29.0	108	15.3	271	21.3
	Smoker	206	36.5	212	30.0	418	32.9
	**Alcohol user**						
	Never	54	9.6	271	38.3	325	25.6
	Stopped	39	6.9	32	4.5	71	5.6
	Drinker	470	83.5	404	57.1	874	68.8
	**Leisure-time physical activity** ^*4^						
	Daily/almost daily	95	16.9	78	11.0	173	13.6
	Once a week or occasionally	131	23.3	54	7.7	185	14.6
	Never	337	59.8	574	81.3	911	71.8
**Morbidity**	**Hypertension status** ^*5^						
	Non-hypertensive	336	59.7	457	64.7	793	62.5
	Untreated	162	28.8	127	18.0	289	22.8
	Uncontrolled	49	8.7	87	12.3	136	10.7
	Controlled	16	2.8	35	5.0	51	4.0
	**Body Mass Index** ^*6^						
	<25 kg/m^2^	309	54.9	373	53.0	682	53.8
	≥25 kg/m^2^	254	45.1	331	47.0	585	46.2
	**Diabetes**						
	No	537	95.4	658	93.2	1195	94.2
	Yes	26	4.6	48	6.8	74	5.8

^*1^Five individuals without information about skin color

*^2^Mean household income of the Census units in the sample (low income < 7 minimum wages, medium income 7–14; high income >14)

*^3^one individual without data about educational level

^*4^one individual without data about the frequency of physical activity

^*5^one individual without data about hypertension treatment

^*6^three individuals without data about their weight and height

The prevalence of measured hypertension (≥140/90 mmHg) or treated hypertension was 38.0 % (95 % CI 34.2 % - 42.0 %). The prevalence was 40.6 % among men (95 % CI 35.9 % - 45.6 %) and 35.8 % among women (95 % CI 31.5 % - 40.5 %), with adjustment for the sampling design. Slightly more than two-thirds of the subjects older than 60 years were hypertensive. The mean age of the hypertensive subjects was 51.6 years (SD = 14.9). The mean of the second SBP measurement was 131.4 mmHg (DP = 21.6), while the average DBP was 81.7 mmHg (DP = 11.9). The uncontrolled hypertensives were older (57.3, SD = 13.8 vs 48,8 SD = 14.8) and more obese (BMI = 28.1 and SD = 5.1 vs BMI = 26.3 and SD = 3.8), in addition to having blood pressure levels higher than the untreated hypertensives (SBP = 159.3 and SD = 20.8 vs 151.5 and SD = 18.9; and DBP = 92.5 and SD = 12.5 vs DBP = 92.0 and SD = 11.3).

Among the hypertensive subjects, only 39 % (29 % of men and 49 % of women) were receiving hypertension treatment, among these subjects, 73 % (75 % of men and 71 % of women) were uncontrolled (BP below 140/90 mmHg).

Regarding the untreated hypertensives, approximately two-thirds (64.4 %) were unaware of their health condition. Among those who were aware of their hypertensive condition but were not under any treatment, slightly more than half (51.5 %) had been treated previously, but abandoned the therapy. The proportion of women who abandoned treatment (26 %) was approximately twice as high as the proportion observed in men (12.4 %).

Cardiovascular diseases represented the main cause of mortality (31.2 %), followed by cancer (24.7 %). Ill-defined causes of death accounted for 4.7 % of the total number of deaths. The deaths occurred outside hospitals (20.6 %) were due to cardiovascular deaths (14), neoplasia (7), external causes (5), ill-defined causes of death (2) and due to a variety to other causes (7). Most cardiovascular deaths were caused by ischemic heart disease (37.7 %), followed by cerebrovascular disease (26.4 %) and hypertension (18.9 %). Among the hypertensive subjects, 40.0 % of deaths were attributed to cardiovascular causes, of which 31.0 % were caused by ischemic heart disease and 31.0 % by cerebrovascular disease. Mortality from cardiovascular deaths was higher among uncontrolled hypertensives than for any other category (Table 2).

**Table 2:** Mortality for causes of death per hypertension category^*1^ – cohort of *Ilha do Governador*, 1991 – 2009

Hypertension status in 1991/92	Causes of death					
	Cardiovascular deaths		Non-cardiovascular deaths		All deaths	
	%	(95 % CI)	%	(95 % CI)	%	(95 % CI)
Non-hypertensive	**1.3**	(0.7 – 2.3)	**7.1**	(5.5 – 9.0)	**8.4**	(6.8 – 10.3)
Untreated	**6.6**	(3.9 – 10.8)	**12.4**	(8.4 – 18.1)	**19.0**	(14.6 – 24.3)
Controlled	**3.6**	(0.7 – 15.4)	**14.0**	(6.7 – 27.0)	**17.6**	(9.8 – 29.6)
Uncontrolled	**15.0**	(9.6 – 22.6)	**15.0**	(9.6 – 22.8)	**30.0**	(21.6 – 40.1)

^*1^Adjusted for the study design

The mean survival time of the non-hypertensives, adjusted for the study design, was 17.5 years (95 % CI 17.3 to 17.7) and it was 16.5 years (95 % CI 16.0 to 17.1), 15.4 years (95 % CI 14.4 to 16.3) and 17.0 years (95 % CI 16.2 to 17.8) for untreated, uncontrolled and controlled hypertensives, respectively.

**Fig. 1:**
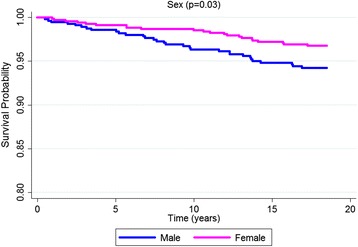
Survival by sex in cohort of adults in *Ilha do Governador*, 1991 – 2009

**Fig. 2:**
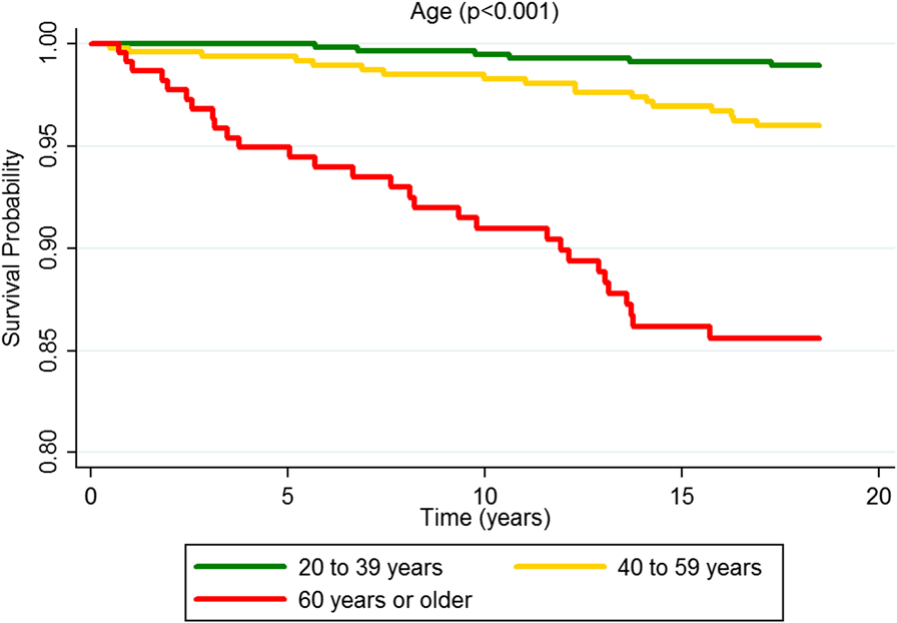
Survival by age in cohort of adults in *Ilha do Governador*, 1991 – 2009

**Fig. 3:**
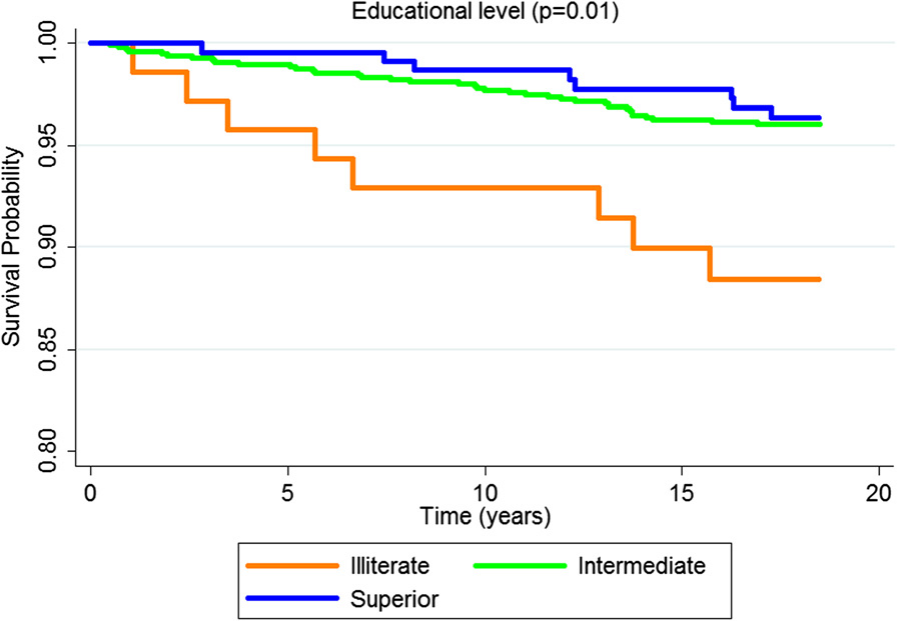
Survival by educational level in cohort of adults in *Ilha do Governador*, 1991 – 2009

**Fig. 4:**
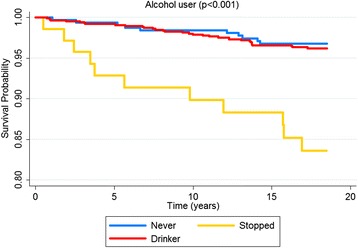
Survival by alcohol user in cohort of adults in *Ilha do Governador*, 1991 – 2009

**Fig. 5:**
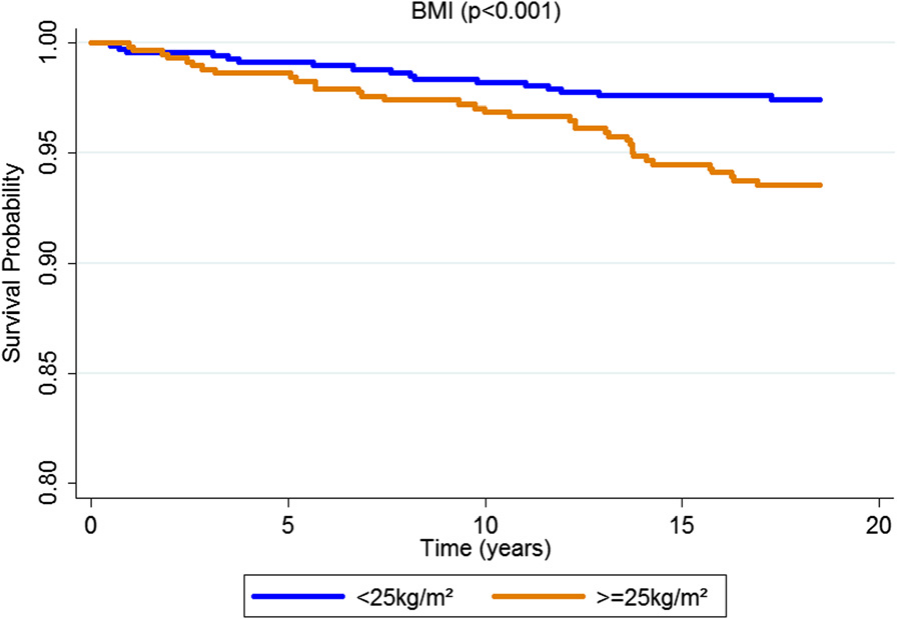
Survival by BMI in cohort of adults in *Ilha do Governador*, 1991 – 2009

**Fig. 6:**
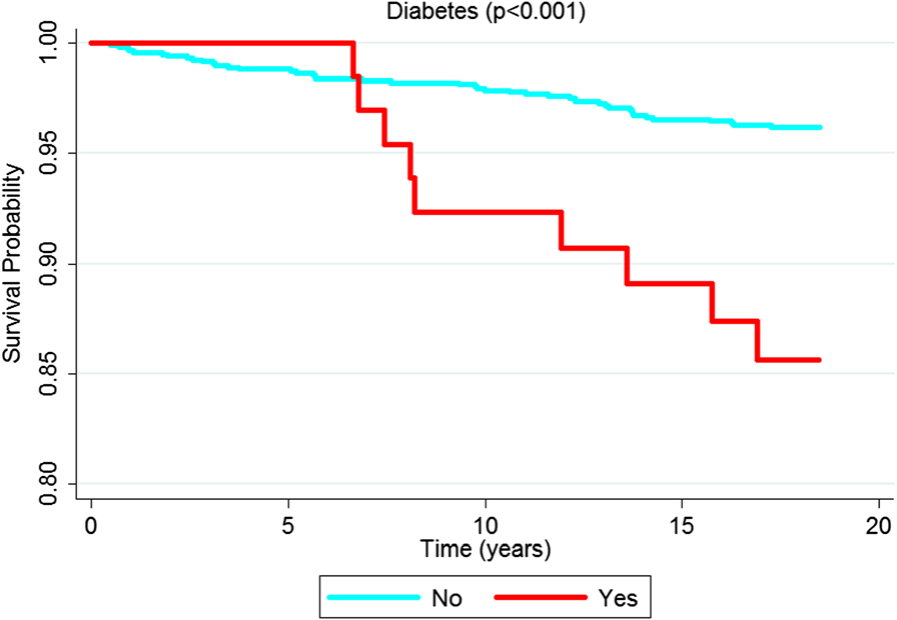
Survival by diabetes in cohort of adults in *Ilha do Governador*, 1991 – 2009

**Fig. 7:**
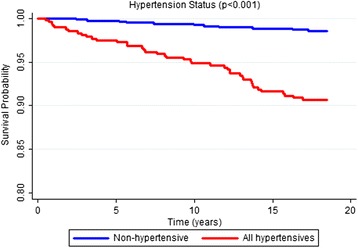
Survival by hypertension status* in cohort of adults in *Ilha do Governador*, 1991 – 2009. *categories of hypertension

**Fig. 8:**
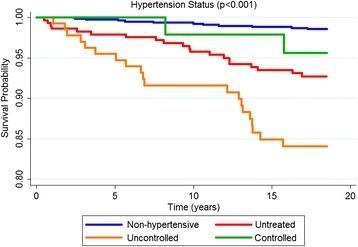
Survival by hypertension status* in cohort of adults in *Ilha do Governador*, 1991 – 2009. *all hypertensive

**Fig. 9:**
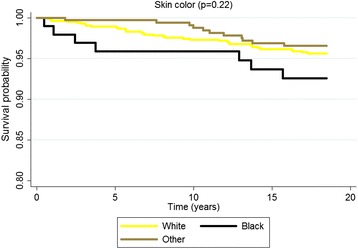
Survival by skin color in cohort of adults in *Ilha do Governador*, 1991 – 2009

According to the Kaplan-Meier curves, men, older subjects, with a lower educational level, who stopped drinking, diabetics and with excessive body weight exhibited shorter survival times. Both untreated and uncontrolled hypertensives exhibited lower survival rates than non-hypertensive or controlled hypertensive subjects. Sex, age, education, alcohol consumption, diabetes, BMI and hypertension were significantly associated with survival rates at *p* < 0.05 for the log-rank test (Figs. 1, 2, 3, 4, 5, 6, 7 and 8). Although there was not a statistically significant difference in survival curves according to skin color black individuals had a shorter survival (Fig. 9).

In the analysis of the association between hypertension and cardiovascular mortality, Cox proportional hazard models were fitted using the non-hypertensive subjects as the reference. Significant effect modifications (additive or multiplicative) were not detected.

**Fig. 10:**
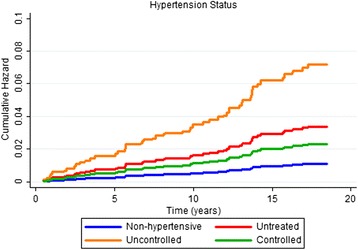
Cumulative hazard for cardiovascular death by hypertension status at 1991/92 in cohort of adults in *Ilha do Governador*, 1991 – 2009

In the final model, when all categories of hypertensive were pooled into a single group, the hazard ratio for cardiovascular death was higher than that of the non-hypertensives and of an intermediate magnitude compared with the categorical analysis (Table 3). The risk of cardiovascular death for both untreated hypertensives and uncontrolled hypertensives was significantly higher than that for non-hypertensives. The risk of cardiovascular death in controlled hypertensives was almost twice as high as that of non-hypertensives, but this difference was not statistically significant (Table 4). The cumulative hazards for cardiovascular death according to hypertension and adjusted by the confounding variables are shown in Fig. 10.

**Table 3:** Hazard ratio for cardiovascular death per unique category of hypertension^*1^ – cohort of *Ilha do Governador*, 1991 – 2009

Hypertension status in 1991/92	HR^*2^	95 % CI^*3^	p-valor
*Non-hypertensive*	*reference*	*-*	*-*
All hypertensives^*4^	3.2	1.6 – 6.7	0.002

^*1^Adjusted for the study design

^*2^HR = hazard ratio

^*3^CI = confidence interval

^*4^Adjusted for sex, age, educational level and body mass index

**Table 4:** Hazard ratios for cardiovascular death per multiple category of hypertension ^*1^ – cohort of *Ilha do Governador*, 1991 – 2009

Hypertension status in 1991/92	HR^*2^	95 % CI^*3^	p-valor
*Non-hypertensive*	*reference*	*-*	*-*
Untreated ^*4^	2.7	1.1 - 6.3	0.03
Controlled ^*4^	2.1	0.4 – 11.5	0.39
Uncontrolled ^*4^	6.1	2.7 - 13.7	<0.00

^*1^Adjusted for the study design

^*2^HR = hazard ratio

^*3^CI = confidence interval

^*4^Adjusted for sex, age, educational level, smoking, leisure-time physical activity and body mass index

## Discussion

The present study showed that the high prevalence of hypertension in the sample of *Ilha do Governador* contributed to an increased risk of cardiovascular death among hypertensive subjects, particularly among the untreated and uncontrolled hypertensive compared with subjects who were non-hypertensive at the time of the cross-sectional study.

Although the clinical diagnosis of hypertension requires repeated measurements on at least two separate occasions and under controlled conditions [2], the diagnosis performed in the cross-sectional study was useful for determining the prognosis of hypertensive. The classification of hypertensive status based on a BP measurement made in a single occasion and on information provided by the patient regarding the use of antihypertensive drugs was able to reveal significant differences in the risk of cardiovascular death over a period of 19 years.

The proportion of deaths from ischemic heart disease was higher than that from cerebrovascular diseases in *Ilha do Governador*. In Brazil, studies showed that cerebrovascular disease is still the more important cause of death among the cardiovascular ones although in some places like São Paulo and Rio de Janeiro the main cause in recent years is ischemic heart disease [17, 18].

We found a risk of cardiovascular death three times higher in hypertensive subjects compared with normotensive. Similar results have been found in others studies [19–23]. The higher hazard ratio for the untreated hypertensive reflects the greater vulnerability to complications in this group and consequently a lower survival time compared with those who receive treatment [24]. A study shown a continuous association between BP and cardiovascular risk, where a reduction of 10 mmHg in the SBP and 5 mmHg in the DBP can reduce the risk of coronary heart disease by 22 % and the risk of stroke by 41 % [25].

Although not significant, the risk of cardiovascular death among controlled hypertensives was low when compared with the other groups of hypertensive. The benefits of antihypertensive therapy in reducing cardiovascular mortality compared with placebo were first reported in longitudinal studies conducted in the 1960s [26, 27].

A review conducted in the early 1960s describing the first studies that demonstrated the long-term benefits of hypertension therapy revealed that the mortality rate decreased by 30-40 % within five years in the treated group compared with the untreated group. This reduction in mortality was attributed to the introduction of drugs such as ganglionic blockers, reserpine, hydralazine and, later, diuretics [28].

Several meta-analyses published in the last decades confirm the efficacy of antihypertensive drugs in the treatment of hypertension as well as their benefits in reducing cardiovascular risk, regardless of age, sex or antihypertensive drug use [25, 29–32].

The antihypertensive treatment may control blood pressure for some individuals but not for all, and, although treatment reduces incidence of cardiovascular outcomes, controlled hypertensive still have higher risk than normotensive [19, 33, 34].

The uncontrolled hypertensive subjects from the *Ilha do Governador* sample were older and exhibited higher BMI values compared with the other groups of hypertensives. Even after the model adjustment for these variables (age and BMI), the magnitude of the cardiovascular risk in the uncontrolled hypertensives was still higher. However, these estimates were imprecise, and it is therefore not possible to state that these differences were significant.

A 30-year follow-up study in Iceland revealed that the risk of cardiovascular mortality among treated and uncontrolled hypertensives was 1.47 times higher than that observed among treated and controlled hypertensive [35]. The interventions of public healthcare programs implemented in the USA in the last decades have prompted a significant increase in the diagnosis, treatment and control of hypertension, which consequently resulted in a reduction in hospitalizations and cardiovascular mortality rates [2]. Increase in prevalence of hypertension could be attributed partly to the aging population, so increase in BP control can significantly reduce cardiovascular mortality.

The treatment of hypertension should not be the only priority of public healthcare policies, which should aim to reduce BP in the entire population by promoting control measures that address proper diet and reductions in salt intake [36–38]. While strategies that focus on the prevention of cardiovascular mortality in high-risk groups are important, the benefits of population-based preventive approaches have more impact in reducing the cardiovascular mortality caused by hypertension [36]. Community*-*based interventions are important for implementing programs that address primary prevention of disease and health promotion to reduce cardiovascular mortality through the control of risk factors [39]. It is important to focus not only on control of individual risk factors but also on improvement of living, working, and environmental conditions, as this can significantly reduce mortality rates due to cardiovascular diseases and their risk factors. This does not mean that the classical risk factors should not be treated, but the fact that this approach is limited, expensive and benefits only a minority of the individuals should be considered [40].

The analysis of the losses in the cross-sectional study [9] suggests that there was no important selection bias due to losses, although the proportion of individuals with higher educational level was larger among the lost ones than among the evaluated individuals, the opposite was seen for the proportion of females. There is no way to evaluate the impact of information bias due to self-reported information about treatment of hypertension but we believe this was not significant as the interviewers were trained to ask the individuals to show all the medication they were taking regularly.

One limitation of the present study is the possible change in the status of exposure and in the other risk factors over time. The *Ilha do Governador* survey was the initial step of a longitudinal study via linkage between databases. However, the risk factors and their levels may have changed over the 19-year interval between the baseline study and the death registry assessment.

Furthermore, despite observed improvements in the quality of the death records in the last decades, the classification of the causes of death may still be incorrect, which would lead to misclassifications [41]. In addition, the number of cardiovascular deaths might be underestimated. A decreasing trend in cardiovascular disease mortality compared with an increased mortality from ill-defined causes was observed in the last decades in the city of Rio de Janeiro, suggesting that these cardiovascular deaths may have been included among the poorly defined causes [17, 42].

Technical problems with the databases from 1991, 1992 and 1996 may have contributed to decrease the sensitivity in identifying true pairs of records in the linkage between the records of the epidemiological survey and the SIM. The amount of missing data may have been greater in those three years than in others because the alternative strategies used to gather the death records were limited to searches in the records from registry offices in the municipality of Rio de Janeiro, unlike other years where the search extended to the entire state of Rio de Janeiro. However, it is more likely that the deaths that occurred shortly after the cross-sectional study have been registered in registry offices near the residential area and would have been identified.

Linkage errors can bias the association measures. When such errors are non-differential and high specificity is maintained, minimally biased risk coefficient estimates are obtained; in general, these values are underestimates [43]. Nevertheless, even probabilistic linkage using properly identified databases is subject to failure in the identification of pairs of records. False-positive results, but not false-negative results, tend to bias the rate ratio toward the null value [13].

## Conclusions

The present study used data from a previous epidemiological survey linked with the Mortality Information System databases to analyze the survival times and cardiovascular death risk of normotensive and treated/untreated, controlled/uncontrolled hypertensive subjects. Despite some limitations, this strategy enabled a longitudinal study on a general population to be conduct at the cost of a cross-sectional study and revealed the importance of hypertension as a predictor of cardiovascular mortality, particularly when blood pressure levels are not controlled.

Diagnosis of hypertension, adherence to treatment and success in controlling this condition represent difficult steps in addressing this important public health problem. It is important to combine preventive strategies targeted at high-risk populations (hypertensive subjects) with other strategies targeted at the general population.
